# Folate Receptor-Targeted Albumin Nanoparticles Based on Microfluidic Technology to Deliver Cabazitaxel

**DOI:** 10.3390/cancers11101571

**Published:** 2019-10-16

**Authors:** Fanchao Meng, Yating Sun, Robert J. Lee, Guiyuan Wang, Xiaolong Zheng, Huan Zhang, Yige Fu, Guojun Yan, Yifan Wang, Weiye Deng, Emily Parks, Betty Y.S. Kim, Zhaogang Yang, Wen Jiang, Lesheng Teng

**Affiliations:** 1 School of Life Sciences, Jilin University, Changchun, Jilin 130012, China; jlujoshuajones@gmail.com (F.M.); sunyating19@gmail.com (Y.S.); lee.1339@osu.edu (R.J.L.); wanggy19@sina.com (G.W.); xiathk8w@gmail.com (X.Z.); jluzhanghuan@gmail.com (H.Z.); 2 Department of Pharmaceutics, College of Pharmacy, The Ohio State University, Columbus, OH 43210, USA; 3 Department of Pharmaceutical Sciences, St. John’s University, Queens, NY 11439, USA; yige.fu15@stjohns.edu; 4 School of Pharmacy, Nanjing University of Chinese Medicine, Nanjing, Jiangsu 210023, China; yanguojun@njucm.edu.cn; 5 Department of Radiation Oncology, The University of Texas Southwestern Medical Center, Dallas, TX 75390, USA; yifan.wang@utsouthwestern.edu (Y.W.); weiye.deng@utsouthwestern.edu (W.D.); eparks@andrew.cmu.edu (E.P.); 6 Department of Neurosurgery, The University of Texas MD Anderson Cancer Center, Houston, TX 77030, USA; bykim@mdanderson.org

**Keywords:** folate receptor, albumin nanoparticle, microfluidic, cabazitaxel

## Abstract

Microfluidic technology (MF) has improved the formulation of nanoparticles (NPs) by achieving uniform particle size distribution, controllable particle size, and consistency. Moreover, because liquid mixing can be precisely controlled in the pores of the microfluidic chip, maintaining high mixing efficiency, MF exerts higher of NP encapsulation efficiency (EE) than conventional methods. MF-NPs-cabazitaxel (CTX) particles (MF-NPs-CTX) were first prepared by encapsulating CTX according to MF. Folate (FA)- Polyethylene glycol (PEG)-NPs-CTX particles (FA-PEG-NPs-CTX) were formulated by connecting FA to MF-NPs-CTX to endow NPs with targeted delivery capability. Accordingly, the mean particle size of FA-PEG-NPs-CTX increased by approximately 25 nm, as compared with MF-NPs-CTX. Upon morphological observation of FA-PEG-NPs-CTX and MF-NPs-CTX by transmission electron microscopy (TEM), all NPs were spherical and particle size distribution was uniform. Moreover, the increased delivery efficiency of CTX in vitro and its strong tumor inhibition in vivo indicated that FA-PEG-NPs-CTX had a powerful tumor-suppressive effect both in vitro and in vivo. In vivo imaging and pharmacokinetic data confirmed that FA-PEG-NPs-CTX had good drug delivery efficiency. Taken together, FA-PEG-NPs-CTX particles prepared using MF showed high efficient and targeted drug delivery and may have a considerable driving effect on the clinical application of targeting albumin NPs.

## 1. Introduction

After the Food and Drug Administration (FDA) approved Abraxane (Abraxis BioScience, Los Angeles, CA, USA) in 2010, albumin NPs have increasingly been attracting researchers’ attention [1]. Nano-formulation delivery systems have been widely used because of their unique biocompatibility and stability [2]. Moreover, sufficient drug binding sites are contained in human serum albumin (HSA) molecules, endowing albumin NPs with a high drug-loading capacity [3,4,5].

Albumin NPs have been exploited to deliver various drugs, such as antineoplastic agents, gene drugs, peptide drugs, and hormone drugs [6,7]. NPs have been prepared by emulsification, desolvation, or nab-technology, but disadvantages included toxic reagent residues, complicated preparation processes, and poor repeatability between batches. Recently, excellent progress has been made in preparing NPs based on MF technology [8,9]. MF enables precise manipulation of liquid flow in the micron size range, allowing the organic and aqueous phases to be rapidly mixed to produce NPs with uniform particle size distribution, high drug encapsulation efficiency (EE), and small batch-to-batch variation.

**Figure 1:**
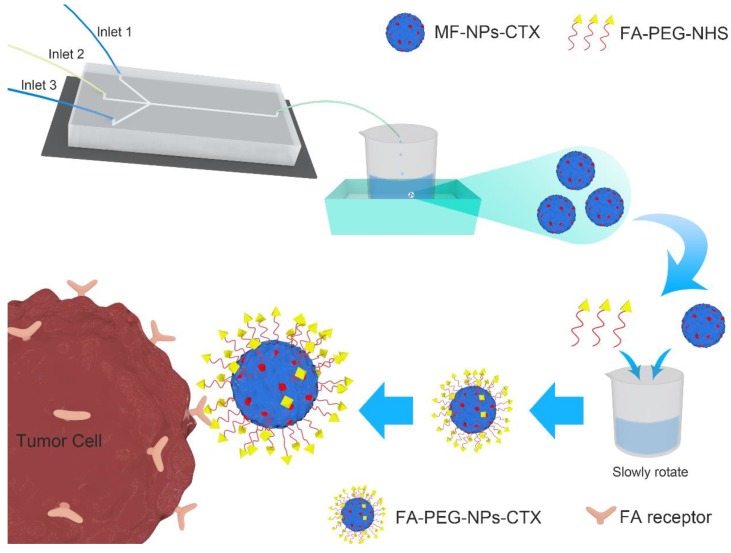
Preparation and function of FA-PEG-NPs-CTX. The aqueous phase was injected through the microfluidic chips at inlet 1 and inlet 3, and the organic phase was injected from inlet 2. MF-NPs-CTX were formed by high shear force mixing in the chip. Folate- Polyethylene glycol -NHS (FA-PEG-NHS) was allowed to react with -NH_2_ of MF-NPs-CTX to form FA-PEG-NPs-CTX. FA-PEG-NPs-CTX were then taken up by tumor cells through FR-mediated endocytosis.

Normally, conventional albumin NPs can only passively deliver drugs to tumor sites through the enhanced permeability and retention (EPR) effect, which severely limits their delivery efficiency. Because many malignant tumor cells express highly specific receptors such as human epidermal growth factor receptor 2 (HER2) [10], transferrin receptor (TfR) [8], and FA receptor (FR) [11], albumin NPs can be chemically modified with the corresponding ligands to provide targeted tumor delivery(Figure 1). FR is highly expressed on the surface of various tumor cells. Additionally, its ligand FA is not immunogenic and has high structural stability and a strong affinity with FR. NPs linked with FA can specifically bind to FR and enter the cytoplasm by endocytosis.

## 2. Results

### 2.1. Characterization

The in vitro characterization of MF-NPs-CTX, FA-PEG-NPs-CTX, and traditionally-prepared NPs (Tr-FA-PEG-NPs-CTX) is shown in Table 1: after FA-PEG attachment, the average of the NP particle size increased by about 25 nm. Because of FA-PEG’s negative charge, FA-PEG-NPs-CTX’s ζ-potential increased from −20.3 ± 0.5 mV to −27.8 ± 0.6 mV. During the FA-PEG-NHS - MF-NPs-CTX reaction, some CTX leaked, and the EE and drug-loading capacity (EC) of FA-PEG-NPs-CTX were both decreased. While polymer dispersity index (PDI) showed no obvious change, FA-PEG-NPs-CTX particles were still uniformly distributed.

**Figure 2:**
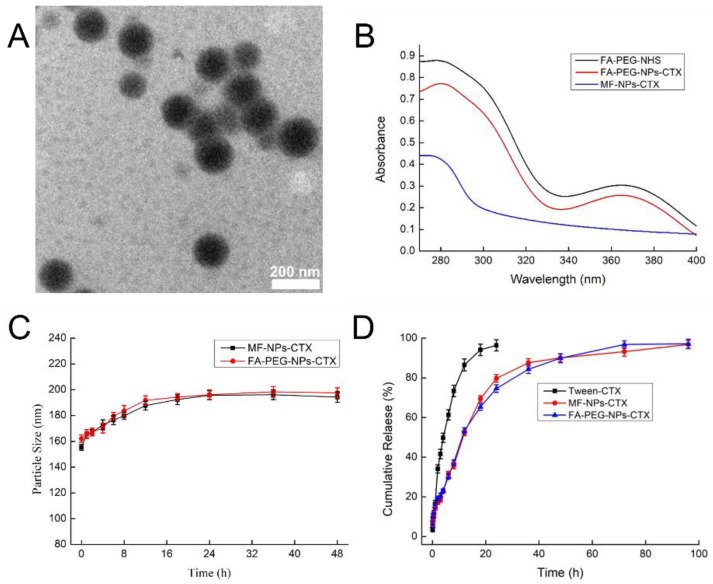
In vitro properties of MF-NPs-CTX and FA-PEG-NPs-CTX. (**A**) FA-PEG-NPs-CTX were observed by TEM. (**B**) The absorption curve of FA-PEG-NHS, FA-PEG-NPs-CTX, and MF-NPs-CTX determined by Ultraviolet–visible spectroscopy (UV-vis). (**C**) Stability of MF-NPs-CTX and FA-PEG-NPs-CTX in vitro. MF-NPs-CTX and FA-PEG-NPs-CTX were dissolved in 10% fetal bovine serum (FBS) (*n* = 3) and placed in a constant temperature shaker at 37 °C, 150 rpm; the particle sizes were measured by Zetasizer Nano particle size analyzer. (**D**) In vitro release of Tween-CTX, MF-NPs-CTX, and FA-PEG-NPs-CTX (*n* = 3). Release solution is 0.1% v/v Tween 80/PBS (pH 7.4).

The morphology of FA-PEG-NPs-CTX (Figure 2A) was observed by transmission electron microscopy (TEM); particles were spherical and uniformly distributed. FA-PEG-NPs-CTX and FA-PEG-NHS had a unique absorption peak at 366 nm (Figure 2B). The FA content of FA-PEG-NPs-CTX was measured after trypsin treatment and found to be 9.28 μg/mg, while the connection efficiency of FA-PEG-NHS was 22.8%. The stability of MF-NPs-CTX and FA-PEG-NPs-CTX in a simulated plasma environment (37 °C, 10% of fetal bovine serum, FBS) is shown in Figure 2C. The particle size of the two NPs increased by about 20 nm within 8 h, and then stabilized. Within 48 h, the average particle size was increased by about 30 nm. These results indicate that MF-NPs-CTX and FA-PEG-NPs-CTX had good stability in serum.

To analyze the cumulative drug release, the preparations were incubated with PBS containing 0.1% Tween-80. Released CTX was measured by HPLC at fixed time points. The in vitro release results of Tween-CTX (CTX was formulated in Tween 80 and ethanol mixed solution according to JEVTANA^®^, 13% ethanol: Tween 80 = 4:1), MF-NPs-CTX, and FA-PEG-NPs-CTX are shown in Figure 2D. CTX was slowly released from MF-NPs-CTX and FA-PEG-NPs-CTX but not from Tween-CTX. Through the release profiles of CTX in MF-NPs-CTX and FA-PEG-NPs-CTX, FA-PEG linkage did not affect the release of FA-PEG-NPs-CTX. Both MF-NPs-CTX and FA-PEG-NPs-CTX had slow drug release behavior. After reaching the tumor site, CTX was gradually released from NPs and killed the tumor cells.

### 2.2. Cell Viability Assay

**Figure 3:**
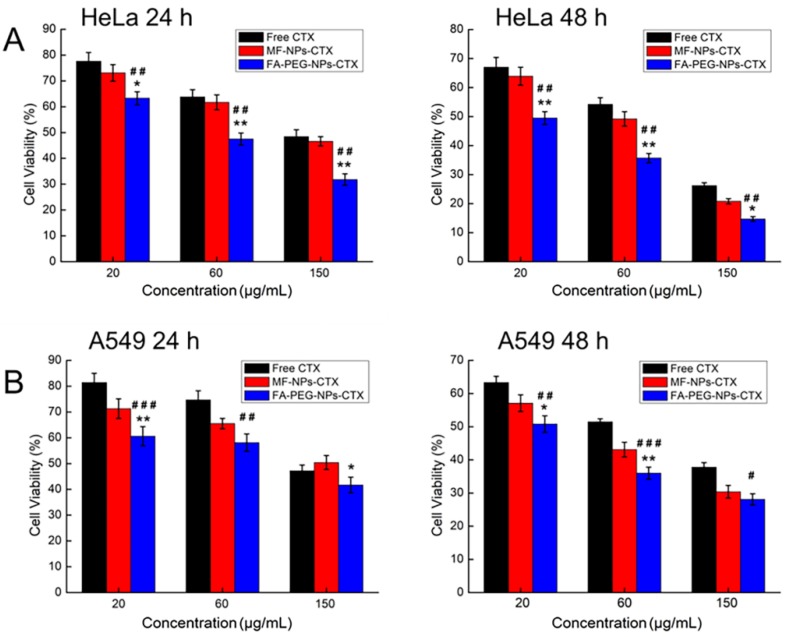
Evaluation FA-PEG-NPs-CTX cytotoxicity by HeLa and A549 cells (*n* = 5). (* *p <* 0.05, ** *p <* 0.01, Student’s *t*-test, FA-PEG-NPs-CTX versus MF-NPs-CTX. ^##^ *p <* 0.01, ^###^ *p <* 0.001, Student’s *t*-test, FA-PEG-NPs-CTX versus Free CTX).

The cytotoxicity of FA-PEG-NPs-CTX was evaluated in both HeLa and A549 cells (Figure 3). Free CTX, MF-NPs-CTX, and FA-PEG-NPs-CTX killed cells in both dose- and time-dependent manners. Moreover, the vectors without CTX (MF-NPs and FA-PEG-NPs) were almost non-toxic to HeLa and A549 cells (Figure S1), thus, we inferred that FR could mediate its uptake by HeLa cells, FA-PEG-NPs-CTX was more cytotoxic to these cells than MF-NPs-CTX. Because of the targeted delivery of CTX by FA-PEG-NPs-CTX and higher EE, FA-PEG-NPs-CTX was more toxic for both cell types than Free CTX.

### 2.3. Cellular Uptake Assay

**Figure 4:**
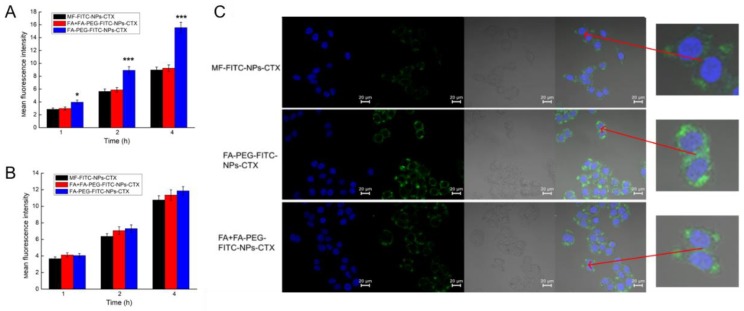
Detection of FA-PEG-FITC-NPs-CTX cellular uptake in vitro. Flow cytometry was used to quantitatively detect the uptake of FITC-labeled FA-PEG-NPs-CTX by HeLa (**A**) and A549 (**B**) (*n* = 3) cells, (* *p* < 0.05, *** *p <* 0.001, Student’s *t*-test, FA-PEG-FITC-NPs-CTX versus MF-FITC-NPs-CTX). (**C**) LCSM was used to qualitatively observe the effect of FA block on the uptake of the CTX-loaded formulation by HeLa cells (×400); the rightmost side was an enlarged view of the area indicated by the arrow. The nucleus was dyed by 4’,6-diamidino-2-phenylindole (DAPI) (blue). FA-PEG-FITC-NPs-CTX and MF-FITC-NPs-CTX were labelled green.

Flow cytometry was used to analyze the cellular uptake of FA-PEG-NPs-CTX labeled by fluorescein isothiocyanate (FITC) (FA-PEG-FITC-NPs-CTX) (Figure 4). The uptake of FA-PEG-FITC-NPs-CTX by HeLa (Figure S2A) and A549 (Figure S2B) cells increased over 1–4 h, and the uptake of FA-PEG-FITC-NPs-CTX by HeLa was 2.73 times that of MF-FITC-NPs-CTX within 4 h (Figure 4A,B). After FA was blocked, the uptake of FA-PEG-FITC-NPs-CTX by HeLa cells was not significantly different from that of MF-FITC-NPs-CTX. To some extent, the uptake of FA-PEG-NPs-CTX by HeLa cells was enhanced by FR mediation. For A549 cells with low FR expression, the uptake of FA-PEG-FITC-NPs-CTX and MF-FITC-NPs-CTX only increased over time. At the same time point, no significant difference was observed in the uptake of A549 cells.

Laser scanning confocal microscope (LCSM) was used to qualitatively observe the effect of FA block on the uptake of NPs by HeLa cells (Figure 4C). The fluorescence intensity of cells treated with FA-PEG-FITC-NPs-CTX after 4 h was significantly stronger than that those treated with MF-FITC-NPs-CTX, and no significant differences in fluorescence intensity were observed between the FA-PEG-FITC-NPs-CTX treatment group and the MF-FITC-NPs-CTX treatment group after FA block. The uptake of FA-PEG-FITC-NPs-CTX by HeLa cells was enhanced by FR mediation.

### 2.4. Biosafety Evaluation

The biosafety of FA-PEG-NPs-CTX was evaluated by hemolytic assay. The erythrocyte hemolysis rate results are shown in Table 2. Because of the strong hemolytic activity of the cosolvent Tween 80 in Tween-CTX, when the CTX concentration was 5.0 μg/mL in the Tween-CTX treatment group, 59.37 ± 0.88% hemolytic cells were counted, and the amount of hemolysis increased as the CTX concentration also increased. For MF-NPs-CTX and FA-PEG-NPs-CTX treatment groups, cell hemolysis rates were all less than 5.0%, in the range of 5.0–200.0 μg/mL. MF-NPs-CTX and FA-PEG-NPs-CTX showed good biosafety results and could be safely administered intravenously.

### 2.5. In Vivo Distribution Experiment

**Figure 5:**
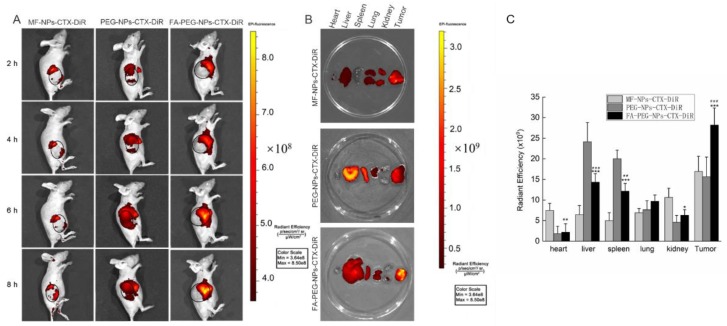
Biodistribution of MF-NPs-CTX-DiR, PEG-NPs-CTX-DiR, and FA-PEG-NPs-CTX-DiR. (**A**) In vivo distribution of HeLa tumor-bearing nude mice at 2, 4, 6, and 8 h after injection via the tail vein with three nanoparticle types with the same DiR content (*n* = 3). (**B**) Distribution of MF-NPs-CTX-DiR, PEG-NPs-CTX-DiR, and FA-PEG-NPs-CTX-DiR in organs and tumors of tumor-bearing nude mice after 8 h. (**C**) Ex vivo radiant fluorescence efficiency at organs and tumor tissues (* *p <* 0.05, ** *p* < 0.01, *** *p <* 0.001, Student’s *t*-test, FA-PEG-NPs-CTX-DiR versus MF-NPs-CTX-DiR, ^##^ *p* < 0.01, ^###^ *p <* 0.001, Student’s *t*-test, FA-PEG-NPs-CTX-DiR versus PEG-NPs-CTX-DiR).

The results of 1,1-dioctadecyl-3,3,3,3-tetramethylindotricarbocyanine iodide (DiR) content for MF-NPs-CTX-DiR, PEG-NPs-CTX-DiR, and FA-PEG-NPs-CTX-DiR after tail vein injection are shown in Figure 5. The fluorescence intensity of MF-NPs-CTX-DiR, PEG-NPs-CTX-DiR, and FA-PEG-NPs-CTX-DiR increased over time at the tumor site (Figure 5A), indicating that both particle types gradually accumulated near the tumor tissue because of the EPR effect. Moreover, FA-PEG-NPs-CTX-DiR had stronger fluorescence intensity in tumor tissues than MF-NPs-CTX-DiR and PEG-NPs-CTX-DiR. The distribution of fluorescence intensity in organ tissues and tumor tissues after euthanasia are shown in Figure 5B. The fluorescence intensity of tumor tissues in the FA-PEG-NPs-CTX-DiR treatment group was much stronger than that in the MF-NPs-CTX-DiR or PEG-NPs-CTX-DiR treatment group (Figure 5C). Furthermore, FA-PEG-NPs-CTX could be directly localized to the tumor site after targeted chemical modification.

### 2.6. In Vivo Therapeutic Efficacy of FA-PEG-NPs-CTX

**Figure 6:**
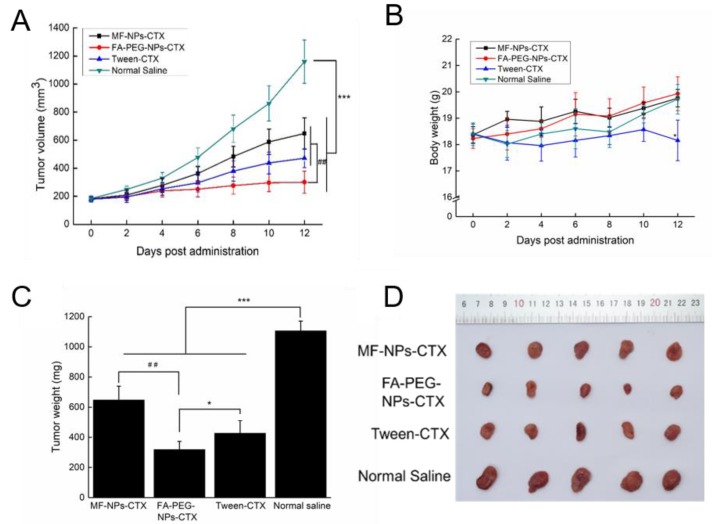
In vivo antitumor effect of MF-NPs-CTX, FA-PEG-NPs-CTX, and Tween-CTX. (**A**) Tumor volume change after 4 days of injection in different administration groups (*** *p <* 0.001, Student’s *t*-test, Tween-CTX, MF-NPs-CTX and FA-PEG-NPs-CTX versus Normal saline. ^##^ *p <* 0.01, Student’s *t*-test, FA-PEG-NPs-CTX versus MF-NPs-CTX and Tween-CTX). (**B**) Bodyweight curves of different treatment groups (*n* = 5) (* *p <* 0.05, Student’s *t*-test, FA-PEG-NPs-CTX versus Tween-CTX). (**C**) Tumor weight of different groups after treatment (*** *p <* 0.001, Student’s *t*-test, Tween-CTX, MF-NPs-CTX and FA-PEG-NPs-CTX versus Normal saline. * *p <* 0.05, Student’s *t*-test, FA-PEG-NPs-CTX versus Tween-CTX. ^##^ *p <* 0.01, Student’s *t*-test, FA-PEG-NPs-CTX versus Tween-CTX). (**D**) Tumor from different groups.

Once the average tumor volume of HeLa tumor-bearing mice reached 180 mm^3^, the drug was injected via the tail vein. Tween-CTX, MF-NPs-CTX, and FA-PEG-NPs-CTX significantly inhibited tumor growth (*** *p* < 0.001) compared with the saline group (Figure 6). The inhibition rate of FA-PEG-NPs-CTX on tumor growth was 74.1%, which was significantly higher than that of MF-NPs-CTX (44.2%) and Tween-CTX (59.3%) (Figure 6A). Moreover, tumor weight in the FA-PEG-NPs-CTX treatment group was significantly lower than that of the MF-NPs-CTX treatment group (Figure 6C). Additionally, the average tumor volume in the FA-PEG-NPs-CTX treatment group was the smallest of all groups (Figure 6D). During the administration period, the bodyweight of the nude mice in the FA-PEG-NPs-CTX treatment group did not change significantly, but the weight of the Tween-CTX treatment group decreased significantly (Figure 6B). Therefore, Tween-CTX was highly toxic to tumor-bearing nude mice. In general, FA-PEG-NPs-CTX had higher biosafety and also achieved better tumor inhibition. Thus, FA-PEG-NPs-CTX may be used successfully to treat tumors.

### 2.7. Pathological Analysis

**Figure 7:**
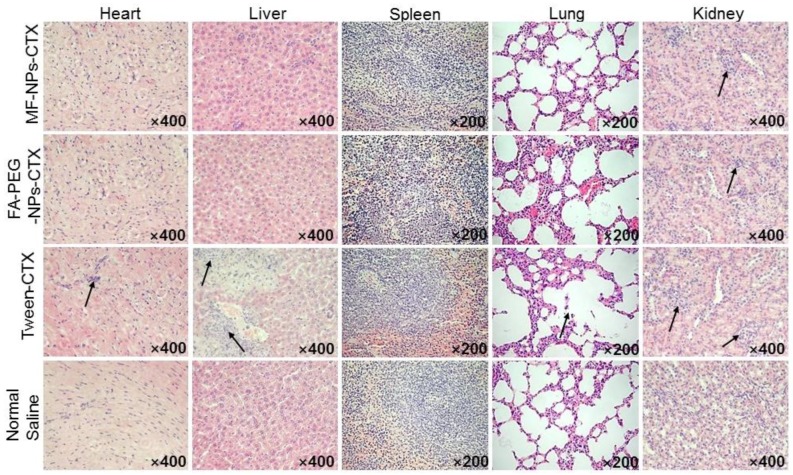
Pathological section analysis of heart, liver, spleen, lung, and kidney by H & E staining after MF-NPs-CTX, FA-PEG-NPs-CTX and Tween-CTX treatment. The arrows indicated the site of the damaged organ.

After multiple administrations, the internal organs of the tumor-bearing nude mice in the Tween-CTX group were shown to be severely damaged (Figure 7): the myocardial fibers were loosely arranged, individual nuclei appeared concentrated, and large areas of hepatocyte necrosis were observed. Additionally, renal tubular epithelial cells showed a high number of eosinophils, and glomerular capillary dilatation was evident. In contrast, only partial lesions appeared in the kidneys of animals from the MF-NPs-CTX and FA-PEG-NPs-CTX treatment groups. Lesions were related to the strong renal toxicity of CTX itself. Thus, FA-PEG-NPs-CTX were shown to have good biosafety.

### 2.8. Plasma Pharmacokinetic Analysis

**Figure 8:**
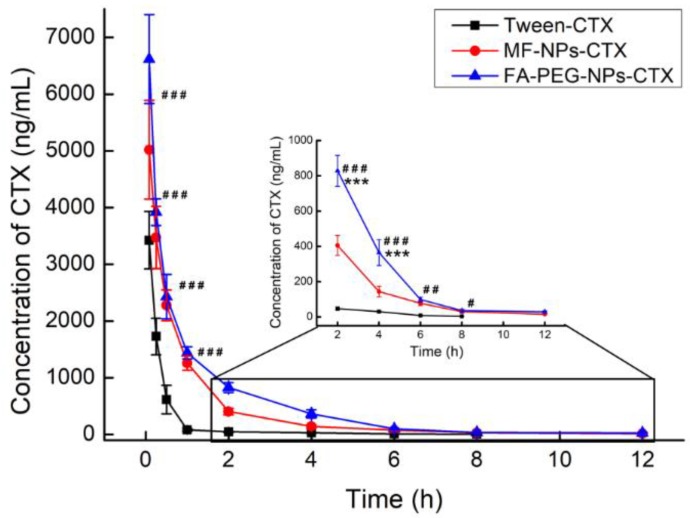
Pharmacokinetic curves of blood concentration of CTX for different drug administration groups (*n* = 5), *** *p* < 0.001, Student’s *t*-test, FA-PEG-NPs-CTX versus MF-NPs-CTX. ^#^ *p* < 0.05, ^##^ *p* < 0.01, ^###^ *p* < 0.001, Student’s *t*-test, FA-PEG-NPs-CTX versus Tween-CTX).

Pharmacokinetic parameters were calculated using the Winnonlin 5.3 software in a non-modal model. After intravenous injection, Tween-CTX was rapidly cleared in plasma (Table 3). Distribution (V) and clearance (CL) volumes were 4.46 times (*** *p* < 0.001) and 4.27 times (*** *p* < 0.001) greater than those of FA-PEG-NPs-CTX, respectively. At the same time, the area under the curve (AUC) of FA-PEG-NPs-CTX was 4.46 times greater than that of Tween-CTX (*** *p* < 0.001). Thus, FA-PEG-NPs-CTX significantly increased the blood concentration of CTX after administration. From the curve of CTX blood concentration over time (Figure 8), FA-PEG-NPs-CTX exhibited the best pharmacokinetic properties after administration of the three-drug preparations via the tail vein.

### 2.9. Pharmacokinetic Analysis of Drug Distribution in Various Tissues and Organs

**Figure 9:**
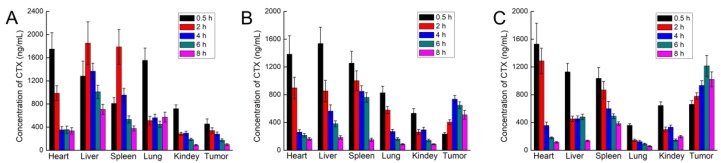
Pharmacokinetic analysis of tissue distribution of CTX at 0.5, 2, 4, 6, 8 h after Tween-CTX (**A**), MF-NPs-CTX (**B**), and FA-PEG-NPs-CTX (**C**) were injected into HeLa tumor-bearing nude mice via the tail vein (*n* = 3).

After Tween-CTX, MF-NPs-CTX, and FA-PEG-NPs-CTX were injected into HeLa tumor-bearing nude mice via the tail vein, CTX distribution was determined in various organs and tumors (Figure 9). After intravenous injection, Tween-CTX was mainly found in the liver, lung, and kidney, while MF-NPs-CTX and FA-PEG-NPs-CTX accumulated in relatively smaller amounts in the liver and lung. In the tumor tissue, as shown in Figure S3, the CTX content of FA-PEG-NPs-CTX in the tumor site gradually increased within 0.5–6 h and reached a higher concentration at 8 h. The tumor site AUC of FA-PEG-NPs-CTX was 3.43 times greater than that of Tween-CTX and 1.75 times greater than that of MF-NPs-CTX. FA-PEG-NPs-CTX exhibited significant tumor-targeted aggregation ability.

## 3. Discussion

Chemotherapy is one of the major strategies used to clinically manage malignant tumors [12,13,14]. As a second-generation taxane, CTX can prevent cell proliferation by binding to tubulin, and because of its low affinity with P-glycoprotein, it can overcome malignant tumor resistance [15,16]. Although CTX has good application prospects for treating malignant tumors, its inability to selectively kill cells leads to severe side effects in patients. Low solubility and side effects of cosolvents also limit the application of CTX. Albumin NPs have certain tumor-targeting properties that can improve in vivo drug distribution and reduce drug toxicity. Although NPs have good biosafety and biocompatibility levels [17,18,19], those prepared by conventional methods present disadvantages such as large batch-to-batch variation, wide particle size distribution, and lack of tumor targeting, which limit their clinical application [20,21]. To solve these problems, we applied microfluidic technology (MF) to encapsulate CTX into NPs [22]. Then, we attached FA-PEG to the NPs as a targeting ligand. FA-PEG had a characteristic absorption at 366 nm, while MF-NPs-CTX did not absorb at this wavelength. Results of the UV-vis absorption comparison and folate content analysis of FA-PEG-NPs-CTX (FA content was 9.28 μg/mg) both indicated that FA-PEG was attached to MF-NPs-CTX. Moreover, because of increased mixing efficiency and shearing force of the organic phase and the aqueous phase in the microfluidic chip, the EE of the prepared nanoparticles was 80% higher than that of the control, and the drug loading was 15% higher. Therefore, the same amount of nanoparticles was swallowed by the cells, and the dose of drugs that enter the cells will be higher, indicating the potential to increase the efficiency of drug entry into cells. The particle size distribution of the NPs was uniform and the PDI < 0.1. Currently, the yield of nanoparticles produced by our MF chip in one batch was around 40%, which is lower than the traditional emulsion method. However, according to our previous studies, MF can significantly improve the uniformity of the nanoparticles. The improved size distribution may further increase drug efficiency. Moreover, to increase the yield by MF, the multi-channel microfluidic chip is being investigated in our lab.

Results of in vitro stability experiments showed that the particle size of MF-NPs-CTX and FA-PEG-NPs-CTX increased by about 30 nm in plasma within 16 h, mainly because of adsorption in plasma of proteins such as opsonin and low-density lipoprotein [23]. However, after 16 h, the particle size tended to stabilize: first, because albumin is part of plasma and is stable in plasma; second, after the drug adsorbed on the nanoparticle’s surface was released, the nanoparticles became more stable [24]. This result could be confirmed by conducting stability experiments. Therefore, we speculate that the NPs were still stable after injection into the bloodstream and could be safely administered through the tail vein.

Results of erythrocyte hemolysis tests showed that when CTX concentration was 5–200 μg/mL, the erythrocyte hemolysis rate of MF-NPs-CTX and FA-PEG-NPs-CTX treatments was still less than 5%, which is in line with the standards. Encapsulation of CTX in the nanoparticles removes the strong hemolysis properties of Tween 80 and improves drug safety[25]. This result confirmed that MF-NPs-CTX and FA-PEG-NPs-CTX had good biosafety levels. The results of in vitro cytotoxicity experiments showed that FA-PEG-NPs-CTX was significantly more cytotoxic to HeLa cells than MF-NPs-CTX at the same CTX concentration. Moreover, cellular uptake experiments by flow cytometry and LCSM showed that the cellular uptake of FA-PEG-NPs-CTX by HeLa and A549 cells were time-dependent. FA blocking profiles indicated that the uptake of FA-PEG-NPs-CTX by HeLa cells was significantly reduced by FA blockage, with no significant difference with the uptake of MF-NPs-CTX. FA blocking had no significant effect on the uptake of A549 cells with low FR expression. Cellular uptake experiments showed that cell-mediated uptake of FA-PEG-NPs-CTX could be enhanced by FR. Combined with cytotoxicity experiments, we concluded that FR mediates endocytosis of more nanoparticles to enhance drug toxicity to cells, possibly leading to more effective treatment in vivo.

Evaluation of the antitumor profile showed that FA-PEG-NPs-CTX have strong tumor-suppressive effects on HeLa-tumor mice with high FR expression. FA-PEG-NPs-CTX’s inhibition rate on tumor growth was 74.1%, which was significantly higher than that of both MF-NPs-CTX (44.2%) and Tween-CTX (59.3%) groups. This was consistent with HeLa cells’ enhanced cellular uptake results. After completing the entire administration course, the weight of mice in the FA-PEG-NPs-CTX and MF-NPs-CTX groups showed little change, while in the Tween-CTX group, the body weight decreased significantly after treatment, showing strong biotoxicity. This was also reflected in the histopathological analysis. FA-PEG-NPs-CTX and MF-NPs-CTX showed targeting effects. Additionally, their aggregation amount in normal tissues and organs was less than that of Tween-CTX, thus, toxicity to normal cells was low. Pharmacokinetic experiments further explored in vivo distribution. Tumor inhibition results showed that nanoparticles were more suitable for treating tumors than Tween-CTX. Additionally, FA-PEG-NPs-CTX had better therapeutic effects than MF-NPs-CTX.

After intravenous injection, the distribution of CTX in vivo was qualitatively and quantitatively examined by imaging and pharmacokinetic experiments, respectively. For in vivo imaging, because the situation of nanoparticles in the body is more complicated, in order to clarify the targeting effect of FA-NPs-CTX-DiR via FR in vivo, the PEG-NPs-CTX-DiR group was added. In vivo imaging showed that MF-NPs-CTX-DiR, PEG-NPs-CTX-DiR, and FA-PEG-NPs-CTX-DiR gradually accumulated at the tumor site after tail vein injection, but FA-PEG-NPs-CTX-DiR showed better accumulation than MF-NPs-CTX-DiR and PEG-NPs-CTX-DiR; thus, we believe this was likely due to the active targeting of FA to the FA-receptor [26]. After 4 h, mice were euthanized to collect the heart, liver, spleen, lung, kidney, and tumors. The fluorescence of FA-PEG-NPs-CTX-DiR at the tumor site was stronger than that of NPs-CTX-DiR, which also indicated that the active targeting of FA to the FA-receptor. Fluorescence also occurred in normal organs because of inevitable reticuloendothelial system (RES) action and renal excretion. Pharmacokinetic experiments were used to quantitatively investigate the distribution of different formulations of CTX in plasma and tissues to explore the reasons why the two nano-formulations were more effective than Tween-CTX. From the CTX plasma pharmacokinetic profile, we could see that its concentration in the FA-PEG-NPs-CTX and MF-NPs-CTX groups decreased less than that of Tween-CTX. The nano-formulations exhibited a slow release of CTX as well as a protective effect on the encapsulated drug. Combined with the distribution of CTX in tissues, we hypothesized two possible reasons for this difference. First, Tween-CTX was rapidly cleared by RES after intravenous injection. In contrast, albumin nano-formulations could only be partially removed by RES because of good biocompatibility and low immunogenicity. Second, during drug absorption, the apparent distribution volume of Tween-CTX was large and was absorbed by whole-body organ tissues; thus, the CTX concentration decreased rapidly at the same time. When CTX was loaded into NPs, the apparent distribution volume was small and the tissue clearance rate was reduced. As such, more NPs were accumulated into the tumor site by the EPR effect. Compared with MF-NPs-CTX, FA-PEG-NPs-CTX had FR targeting, and the modification of PEG prolonged its blood circulation time. This further increased the aggregation of CTX at the tumor site, and thus allowed FA-PEG-NPs-CTX to achieve a better tumor-suppressive effect. We also evaluated the PEGylation effect on the cellular uptake of this ligand-target nanoparticle in vitro. Interestingly, no statistical differences were observed by MTT in either Hela cells (48.7 ± 5.3% versus 46.8 ± 8.3%, MF-NPs-CTX versus MF-PEG-NPs-CTX, 48 h) or A549 cells (44.6 ± 4.6% versus 46.1 ± 7.0%, MF-NPs-CTX versus MF-PEG-NPs-CTX, 48 h) treated with MF-NPs-CTX or MF-PEG-NPs-CTX. The MF-PEG-NPs-CTX was prepared according to the method described in the FA-PEG-NPs-CTX part, with the FA-PEG-NHS replaced with PEG-NHS. This was consistent with our previous finding that FA can significantly improve the targeting effect of the nanoparticles [27]. It is generally believed that PEGylation can significantly increase the nanoparticle systemic circulation time by avoiding rapid clearance by reticuloendothelial system. However, recent studies also indicated that PEGylation may have no direct effect or even inhibit the cellular uptake of ligand-target nanoparticles [28]. Further investigation may shed light on the precise molecular mechanisms involved in the PEGylation effect on cellular uptake of ligand-target nanoparticles.

## 4. Materials and Methods

### 4.1. Materials and Animals

FA-PEG-NHS and MeO-PEG-NHS were purchased from Shanghai Ziqi Biotechnology Co. Ltd (Shanghai, China). CTX was purchased from Jiangsu Taxus Biotechnology Co. Ltd (Jiangsu, China). Carbamazepine was provided by Yuanye Biotechnology Shanghai Co. Ltd (Shanghai, China). Human serum albumin (HSA) was obtained from Octapharma (Vienna, Austria). The microfluidic chip was designed independently and customized at the Dalian Institute of Chemical Physics, Chinese Academy of Sciences (Dalian, China). Dulbecco’s Modified Eagle’s Medium (DMEM), fetal bovine serum (FBS), and penicillin-streptomycin were obtained from Gibco (Gaisburg, MD, USA). Fluorescein isothiocyanate (FITC), 4’,6-diamidino-2-phenylindole (DAPI) and acetonitrile were purchased from Sigma. 1,1-dioctadecyl-3,3,3,3-tetramethylindotricarbocyanine iodide (DiR iodide) was purchased from Thermo Fisher Scientific (Eugene, OR, USA). Tween-CTX was formulated in Tween 80 and ethanol mixed solution according to JEVTANA^®^ (Eugene, OR, USA). The rest of the chemical reagents were of analytical or chromatographic purity.

Cervical cancer (HeLa) and lung cancer (A549) cells were obtained from ATCC and cultured in DMEM medium containing 10% FBS, 100 μg/mL streptomycin, and 100 mg IU/mL penicillin. The cells were cultured at 37 °C in a humidified incubator in 5% CO_2_.

Six to eight weeks old female BALB/c nude mice were purchased from Beijing Vital River Laboratory Animal Technology Co, Ltd. (Beijing, China). Female SD rats (200 g) were purchased from Liaoning Changsheng Biotechnology Co. Ltd. (Benxi, China). All animal procedures were performed in accordance with the Guidelines on Humane Treatment of Laboratory Animals and procedures for the care and use of laboratory animals. All animal experiments were approved by the Institutional Animal Ethics Committee (Jilin University) (number 201810023).

### 4.2. Preparation of FA-PEG-NPs-CTX

According to previous research, the inverted W-type microfluidic chip was designed independently[9] , MF-NPs-CTX were prepared by the microfluidic chip. Specifically, a 20.0 mg/mL CTX ethanol solution and a 12.5% sodium chloride solution were prepared and incubated in a 40 °C water bath with HSA (200 mg/mL). Deionized water was placed in a 60 °C water bath and heated to a constant temperature.

1.0 mL and 10.0 mL syringes were each attached to a microinjection pump: the 1.0 mL syringe was connected to inlet 2 of the microfluidic chip and the 10.0 mL syringes was connected to inlets 1 and 3. The entire microfluidics chip line was cleaned with deionized water before preparing the NPs. Then, 20.0 mg/mL CTX solution, 12.5% NaCl solution, and HSA were mixed at a volume ratio of 2:1:1 and placed in the 1.0 mL syringe, at a flow rate of 40.0 μL/min. Moreover, deionized water was placed in the 10.0 mL syringe at 60 °C with a flow rate of 600.0 μL/min. After mixing in the channel of the inverted W-type passive microfluidics chip, MF-NPs-CTX were obtained from the chip by connecting the tube and rapidly cooling in an ice water bath. MF-NPs-CTX were concentrated through a hollow fiber ultrafiltration column with a molecular weight cut off of 50 kD.

Concentrated MF-NPs-CTX was chemically modified with FA-PEG-NHS, and FA-PEG was attached to the MF-NPs-CTX via an amide bond. The bond was formed by dissolving 50.0 mg of FA-PEG-NHS in 2.0 mL of 2.0 M pH 10.0 Na_2_CO_3_/NaHCO_3_ buffer solution, and slowly adding it dropwise to MF-NPs-CTX, which was also dissolved in 2.0 M pH 10.0 Na_2_CO_3_/NaHCO_3_ buffer solution. The reaction was stirred at room temperature for 2 h. Then, the reacted NP solution was placed in an ultrafiltration centrifuge tube with a molecular weight cut off of 50 kDa and centrifuged at 3214× *g* for 10 min. The solution was repeatedly washed with deionized water to remove unreacted FA-PEG-NHS, concentrated by centrifugation to obtain FA-PEG-NPs-CTX, lyophilized, and set aside.

### 4.3. Characterization

Lyophilized MF-NPs-CTX and FA-PEG-NPs-CTX were re-dissolved with deionized water, and the particle size and ζ-potential were measured by a laser particle size analyzer. EE and EC of MF-NPs-CTX and FA-PEG-NPs-CTX were determined by high-performance liquid chromatography (HPLC) and calculated by the equation below. The morphology of MF-NPs-CTX and FA-PEG-NPs-CTX were observed by TEM (JEOL JEM 2100, Tokyo, Japan). To achieve this, MF-NPs-CTX and FA-PEG-NPs-CTX were dissolved and diluted with deionized water, then added dropwise to a copper mesh, dried, and observed by TEM. To assess the stability of FA-PEG-NPs-CTX in plasma after intravenous injection, the stability of FA-PEG-NPs-CTX was evaluated in a simulated plasma environment. FA-PEG-NPs-CTX were dissolved in 10% FBS and then placed in a constant temperature shaker at 37 °C, 150 rpm. A certain amount of sample was taken out at regular intervals to measure particle size by Zetasizer Nano particle size analyzer (Zetasazer Nano ZS90, Malvern, UK) [29].

(1) $$\mathrm{EE}=\frac{\mathrm{weight}\;\mathrm{of}\;\mathrm{CTX}\;\mathrm{in}\;\mathrm{NPs}}{\mathrm{weight}\;\mathrm{of}\;\mathrm{CTX}\;\mathrm{in}\;\mathrm{NPs}+\;\mathrm{weight}\;\mathrm{of}\;\mathrm{CTX}\;\mathrm{in}\;\mathrm{the}\;\mathrm{filtrate}\;}\times 100\text{\%},$$

(2) $$\mathrm{EC}=\frac{\mathrm{weight}\;\mathrm{of}\;\mathrm{CTX}\;\mathrm{in}\;\mathrm{NPs}}{\mathrm{weight}\;\mathrm{of}\;\mathrm{NPs}}\times 100\text{\%}.$$

To evaluate the in vitro release of the formulation, MF-NPs-CTX and FA-PEG-NPs-CTX with a CTX content of 5.0 mg were accurately weighed (*m*) and dissolved in 2.0 mL of saline. The NPs were then transferred to dialysis bags with a molecular weight cut off of 8000–10,000 Da. In the meantime, 5.0 mg CTX was accurately weighed, dissolved in 2.0 mL of mixed solvent (13% ethanol: Tween 80 = 4:1), transferred to a dialysis bag, and placed in 80 mL of PBS release solution (*V*_0_) that contained 0.1% Tween 80 [30]. The solution and the dialysis bag were set to rotate at 200 rpm, 37 °C. At a specific time point, 1.0 mL of each solution (*V*) was taken to determine CTX concentration (*C_i_*) by HPLC, and 1.0 mL of fresh release solution was added. The cumulative release rate (*Q*%) was calculated by the equation below:

(3) $$Q\;\left(\text{\%}\right)=\frac{V_{0}\times C_{i}+V\times \sum_{i=1}^{i-1}C_{i-1}}{m\times EE}\times 100\text{\%}.$$

### 4.4. Cellular Uptake Assay

The cellular uptake of FA-PEG-NPs-CTX by HeLa and A549 cells was analyzed by laser confocal microscopy. HSA was first labeled with FITC and then dissolved in 25.0 mM Na_2_CO_3_/NaHCO_3_ buffer solution at pH = 9.8 with a concentration of 5.0 mg/mL. FITC was added to the HSA solution with a concentration of 0.1 mg/mL. The solution was incubated overnight at room temperature with constant stirring, and the mixed solution was passed through an ultrafiltration centrifuge tube with a molecular weight cut off of 50 kDa. The solution was centrifuged at 12857× *g* for 10 min, and the concentrate was washed several times with Na_2_CO_3_/NaHCO_3_ buffer solution to obtain FITC-labeled HSA. FA-PEG-FITC-NPs-CTX was prepared by labeled HSA with the same procedure used for FA-PEG-NPs-CTX.

In the FA blockage experiment, cells from the FA-blocked group were cultured in complete medium with an FA content of 5.0 mg/mL one week earlier. HeLa, FA-blocked HeLa, and A549 cells in the logarithmic growth phase were planted at a density of 1.0 × 10^5^ cells/dish and cultured for 24 h. Then, the same FA-PEG-FITC-NPs-CTX protein content was added to each well. After incubating for 1, 2, and 4 h, the medium was removed and washed with cold PBS. Cells were fixed with 4% paraformaldehyde for 15 min. Nuclei were stained with 400.0 μL 1.0 μg/mL DAPI for 10 min, washed, and covered with cold PBS. The difference in fluorescence intensity after uptake of nanoparticles by cells was determined by LCSM.

Flow cytometry was used to analyze the uptake of FA-PEG-NPs-CTX by HeLa and A549 cells. First, both cell types were added at a cell density of 1.0 × 10^5^ cells/well in a 12-well plate. Cells in the FA-blocked group were cultured in complete medium with an FA content of 5.0 mg/mL one week in advance. After the two cell lines were cultured in a 12-well plate for 24 h, the same protein contents of FA-PEG-FITC-NPs-CTX and MF-FITC-NPs-CTX were added to each well. At 1, 2, and 4 h, the cell culture medium was aspirated and cells were washed with PBS. Two hundred microliters of trypsin were then added to each well to digest the cells, and the medium was neutralized and centrifuged at 82× *g* for 5 min. Cells were fixed with 500.0 μL of 4% paraformaldehyde, untreated cells were used as a control, and fluorescence intensity was measured by flow cytometry [31].

### 4.5. Analysis of Surface Folate Content

FA-PEG-NHS was dissolved in a 2.0 M pH = 10.0 Na_2_CO_3_/NaHCO_3_ buffer solution, then diluted to 200.0, 100.0, 50.0, 20.0, 10.0, 5.0, and 2.0 μg/mL. UV-vis (scanning wavelength: 366 nm) was used to determine the absorbance of different FA-PEG-NHS concentrations. The standard curve was drawn by taking concentration *X* (μg/mL) of FA-PEG-NHS as the abscissa and absorbance *Y* as the ordinate. Lyophilized FA-PEG-NPs-CTX (10.0 mg) was dissolved in phosphate buffer solution at pH = 7.4. Then, a certain amount of trypsin was added and digested for 4 h, and the absorbance was measured by UV-vis at 366 nm. MF-NPs-CTX with the same protein content was dissolved in phosphate buffer, and the same procedure was conducted and used as a matrix to perform zero calibration of the absorbance.

### 4.6. Cell Viability Assay

Cells in the logarithmic growth phase were added at a density of 5000–8000 cells/well to 96-well plates. After 24 h, FA-PEG-NPs-CTX, MF-NPs-CTX, and free CTX with CTX concentrations of 20.0, 60.0, and 150.0 μg/mL were added. After 24 h and 48 h, respectively, 10.0 μL of 5.0 mg/mL MTT [3-(4,5-dimethylthiazol-2-yl)-2,5-diphenyltetrazolium bromide] reagent were added to each well and incubated for 4 h. Then, the medium was removed and 100 μL of DMSO was added to dissolve the formazan crystals. Absorbance at 490 nm was read on a microplate reader, and the half-maximal inhibitory concentration (IC50) was calculated by SPASS 17.0 software analysis (SPASS, Chicago, IL, USA) [32].

### 4.7. Hemolytic Evaluation

The biosafety of FA-PEG-NPs-CTX was evaluated via the hemolysis test. Tween-CTX, FA-PEG-NPs-CTX, and MF-NPs-CTX with CTX concentrations of 5.0, 10.0, 20.0, 50.0, 100.0, and 200.0 μg/mL were added to 1.0 mL of 2% mouse erythrocytes in 1.5 mL EP tubes and incubated for 2 h. The saline-treated group and the 1.0% Triton X-100-treated group served as negative and positive controls, respectively. After incubation, the EP tubes were centrifuged at 43× *g* for 5 min, and 100.0 μL of the supernatant were taken to a 96-well plate. The absorbance was measured at 540 nm using a microplate reader. The hemolysis rate was calculated according to the following equation:

(4) $$\mathrm{Hemolysis}\;\mathrm{Rate}=\frac{\mathrm{Absorbance}-\mathrm{Negative}\;\mathrm{Group}}{\mathrm{Positive}\;\mathrm{Group}-\mathrm{Negative}\;\mathrm{Group}}\times 100\text{\%}.$$

### 4.8. In Vivo Distribution Experiment

The in vivo distribution of the nano-formulations was examined by in vivo imaging experiments. MF-NPs-CTX-DiR and FA-PEG-NPs-CTX-DiR were prepared by encapsulating DiR and CTX following the same protocol as that used for MF-NPs-CTX and FA-PEG-NPs-CTX. The preparation method of PEG-NPs-CTX-DiR was the same as that of FA-PEG-NPs-CTX-DiR, except that FA-PEG-NHS was replaced by MeO-PEG-NHS, MF-NPs-CTX-DiR, PEG-NPs-CTX-DiR, and FA-PEG-NPs-CTX-DiR with the same DiR content were injected into nude mice through the tail vein. The nude mice were anesthetized with pentobarbital sodium at 1, 2, 4, and 8 h, and fluorescence distribution was observed using an IVIS in vivo imaging system. The excitation wavelength was 745 nm and the emission wavelength was 820 nm. After 8 h, the nude mice were euthanized to evaluate fluorescence intensity in the heart, liver, spleen, lung, kidney, and tumor tissues.

### 4.9. In Vivo Therapeutic Efficacy of FA-PEG-NPs-CTX

The anti-tumor effect of FA-PEG-NPs-CTX was evaluated using a HeLa tumor-bearing nude mouse model. About 5.0 × 10^6^ HeLa cells were injected subcutaneously into the lower right side of each nude mouse. When the average tumor volume reached 100–200 mm^3^, the nude mice were randomly divided into 4 groups (*n* = 5) for Tween-CTX, MF-NPs-CTX, FA-PEG-NPs-CTX, and saline treatment. On days 0, 4, and 8, the nude mice were injected with a CTX dose of 2.0 mg/kg via the tail vein. The body weight of the mice in each group and the length and width of the tumors were measured every 2 days. On day 12, five mice from each group were euthanized to harvest heart, liver, spleen, lung, kidney, and tumor tissues.

### 4.10. Pathological Section Analysis

Heart, liver, spleen, lung, and kidney obtained in “4.9” were fixed with 4% paraformaldehyde and analyzed by H & E staining to determine whether the drug preparation had damaged the internal organs of the nude mice[33].

### 4.11. Plasma Pharmacokinetic Analysis

Pharmacokinetic studies were performed by using SD rats of about 300 g that were randomly divided into 3 groups (*n* = 5). Tween-CTX, MF-NPs-CTX, and FA-PEG-NPs-CTX were injected into the tail veins at a dose of 5.0 mg/kg. At 0.083, 0.25, 0.5, 1, 2, 4, 6, 8, and 12 h after administration, about 500 μL of blood were taken from the orbital venous plexus, placed in a previously prepared EP tube treated with heparin sodium, and centrifuged at around 13,000× *g* for 5 min. The supernatant was taken to obtain plasma. The plasma sample was measured by UPLC-MS/MS, and the peak area was input into the standard curve of the CTX plasma sample to calculate the CTX concentration in the plasma sample. The curve was obtained by taking time *X* (h) after administration as the abscissa and plasma concentration *Y* (ng/mL) of CTX as the ordinate. Pharmacokinetic parameters were calculated by using the Winnonlin 5.3 software (CERTARA, Princeton, NJ, USA) in a non-modal model.

### 4.12. Pharmacokinetic Analysis of Drug Distribution in Various Tissues and Organs

Pharmacokinetics were analyzed to investigate drug accumulation in various tissues and organs of HeLa tumor-bearing nude mice after tail vein injection. Forty-five HeLa tumor-bearing nude mice were randomly divided into three groups of 15 rats each. Tween-CTX, MF-NPs-CTX, and FA-PEG-NPs-CTX were injected through the tail vein at a dose of 2.0 mg/kg. Three nude mice were euthanized from each group at 0.5, 2, 4, 6, and 8 h to harvest heart, liver, spleen, lung, kidney, and tumor tissues. Organs and tumor tissues were weighed and homogenized by adding two times the weight of saline. Tissue homogenates were sampled by UPLC-MS/MS by the established CTX tissue content analysis method.

### 4.13. Statistic and Analysis

Data analysis was performed using the SPSS 17.0 software (SPASS, Chicago, IL, USA), and all chart data were expressed as mean ± standard error (Mean ± SD). Significance was calculated by Student’s *t*-test. When * *p* < 0.05 or ^#^ *p* < 0.05, a statistical difference between the two groups of data was noted. When ** *p* < 0.01 or ^##^ *p* < 0.01, a significant difference between the two groups of data was noted. When *** *p* < 0.001 or ^###^ *p* < 0.01, a highly significant difference between the two sets of data was noted.

## 5. Conclusions

Albumin NPs were prepared in a self-assembled manner based on MF technology (MF-NPs-CTX), and, accordingly, the inverted W-type microfluidic chip was designed independently. Our results indicated that FA-PEG-NPs-CTX had a significantly stronger tumor-suppressive effect with high FR expression both in vitro and in vivo. The modification of FA-PEG not only enhances NP tumor-targeting but also prolongs blood circulation time to some extent. We believe that targeted NPs prepared by MF technology have broad application prospects in clinically treating patients with cancer.

## Figures and Tables

**Table 1:** Characterization of FA-PEG-NPs-CTX vitro properties (*n* = 3).

Sample Name	Size (nm)	ζ-potential (mV)	PDI	EE (%)	EC (%)
MF-NPs-CTX	139.8 ± 2.6	−20.3 ± 0.5	0.093 ± 0.061	84.5 ± 1.8	15.7 ± 0.4
FA-PEG-NPs-CTX	162.4 ± 3.1	−27.8 ± 0.6	0.102 ± 0.032	81.3 ± 1.6	15.3 ± 0.3
Tr-FA-PEG-NPs-CTX	168.8 ± 8.3	−28.6 ± 0.9	0.248 ± 0.31	49.8 ± 0.6	5.2 ± 0.8

**Table 2:** Cell hemolysis rate results (*n* = 3).

Sample Name	5.0 μg/mL	10.0 μg/mL	25.0 μg/mL	50.0 μg/mL	100.0 μg/mL	200.0 μg/mL
MF-NPs-CTX	0.33 ± 0.07	0.46 ± 0.12	0.68 ± 0.12	2.15 ± 0.17	3.67 ± 0.28	4.37 ± 0.36
FA-PEG-NPs-CTX	0.36 ± 0.09	0.65 ± 0.13	0.73 ± 0.14	2.54 ± 0.16	3.35 ± 0.23	4.14 ± 0.45
Tween-CTX	59.37 ± 0.88	70.17 ± 0.67	78.23 ± 1.37	83.47 ± 1.42	89.95 ± 1.46	94.9 ± 1.27

**Table 3:** Pharmacokinetic parameters of CTX after intravenous injection of different drugs (*n* = 5).

Parameters	Tween-CTX	MF-NPs-CTX	FA-PEG-NPs-CTX
C_max_ (ng/mL)	3426.00 ± 476.52	4573.33 ± 537.41	6618.38 ± 783.66
AUC (ng/mL/h)	1419.07 ± 120.61	4688.60 ± 489.83 ^###^	6329.06 ± 776.64 ***
V (mL)	1990.87 ± 179.36	635.39 ± 137.45 ^###^	446.03 ± 80.51 ***
CL (mL/h)	832.72 ± 131.41	254.13 ± 35.83 ^###^	194.96 ± 38.47 ***

(*** *p* < 0.001, Student’s *t*-test, FA-PEG-NPs-CTX versus Tween-CTX. ^###^ *p* < 0.001, Student’s *t*-test, MF-NPs-CTX versus Tween-CTX).

## Supplementary Materials

The following are available online at https://www.mdpi.com/2072-6694/11/10/1571/s1, Figure S1: Cytotoxicity study with vector (MF-NPs and FA-PEG-NPs), Figure S2: LCSM was used to qualitatively detect the uptake of FA-PEG-FITC-NPs-CTX by HeLa (A) and A549 (B) cells at 1, 2, and 4 h, Figure S3: Distribution of CTX in tumor tissues after tail vein injection of Tween-CTX, MF-NPs-CTX, and FA-PEG-NPs-CTX (*n* = 3).
