# Supplementary material for: Folate Receptor-Targeted Albumin Nanoparticles Based on Microfluidic Technology to Deliver Cabazitaxel
Source: Cancers (Basel). 2019 Oct 16;11(10):1571. doi: 10.3390/cancers11101571 (PMC6827099; doi:10.3390/cancers11101571)
Supplement: Supplementary file 1 [file cancers-11-01571-s001.pdf]

Supplementary Materials: Folate Receptor-Targeted Albumin Nanoparticles Based on Microfluidic Technology to Deliver Cabazitaxel

Fanchao Meng, Yating Sun, Robert J. Lee, Guiyuan Wang, Xiaolong Zheng, Huan Zhang, Yige Fu, Guojun Yan, Yifan Wang, Weiye Deng, Emily Parks, Betty Y.S. Kim, Zhaogang Yang, Wen Jiang and Lesheng Teng

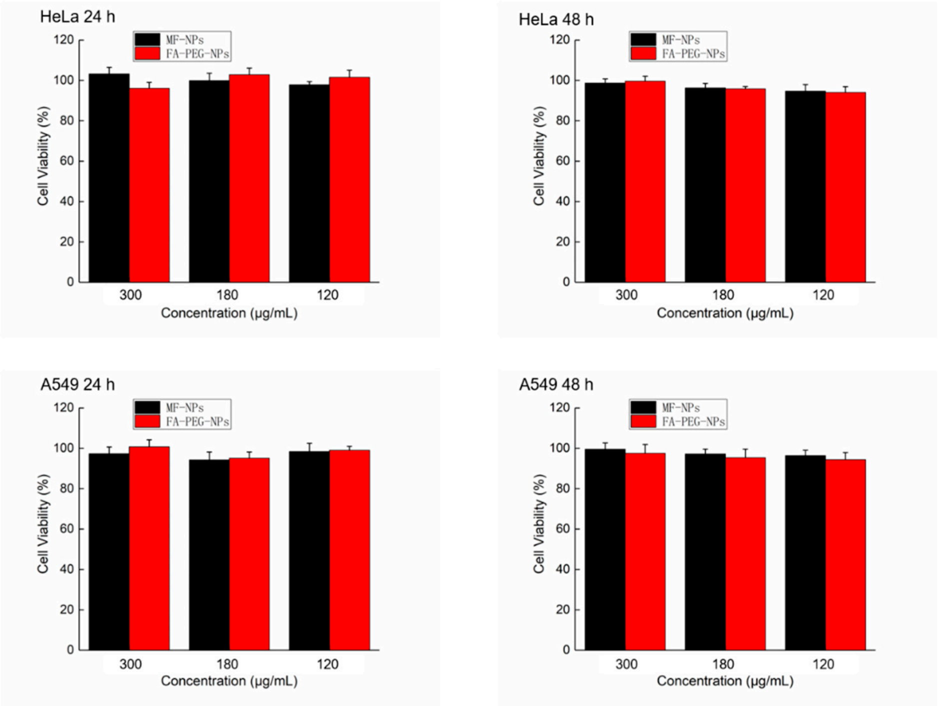

Figure S1. Cytotoxicity study with vector (MF-NPs and FA-PEG-NPs). Evaluation of MF-NPs and FA-PEG-NPs cytotoxicity by HeLa and A549 cells ($n = 5$).

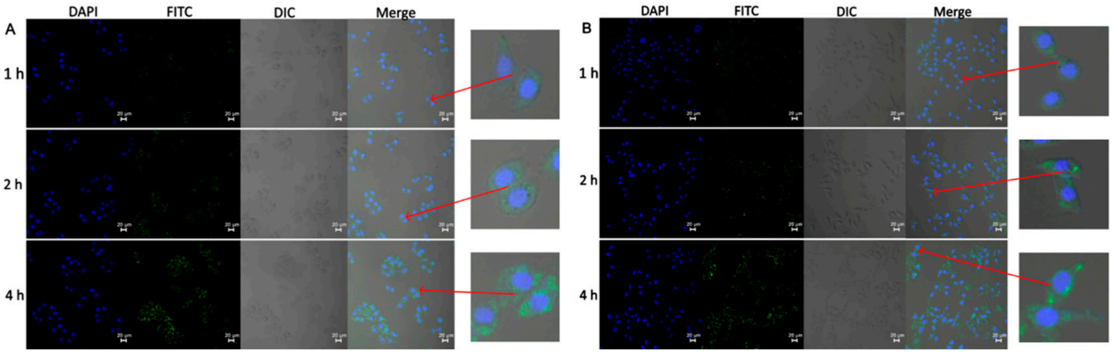

Figure S2. LCSM was used to qualitatively detect the uptake of FA-PEG-FITC-NPs-CTX by HeLa (**A**) and A549 (**B**) cells at 1, 2, and 4 h. The nucleus was dyed blue by DAPI and green was dyed by FA-PEG-FITC-NPs-CTX and MF-FITC-NPs-CTX. The rightmost side was an enlarged view of the area indicated by the arrow.

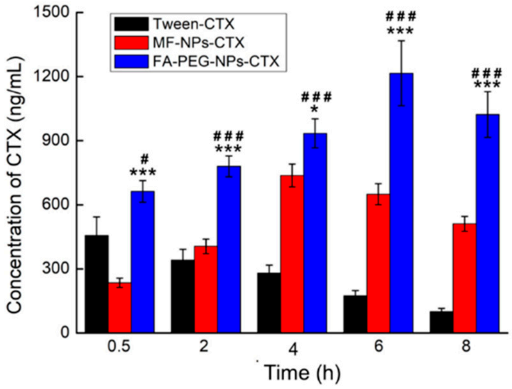

Figure S3. Distribution of CTX in tumor tissues after tail vein injection of Tween-CTX, MF-NPs-CTX, and FA-PEG-NPs-CTX ($n = 3$) (* $p < 0.05$, *** $p < 0.001$, Student's t-test, FA-PEG-NPs-CTX versus MF-NPs-CTX, # $p < 0.05$, ### $p < 0.001$, Student's t-test, FA-PEG-NPs-CTX versus Tween-CTX).

© 2019 by the authors. Licensee MDPI, Basel, Switzerland. This article is an open access article distributed under the terms and conditions of the Creative Commons Attribution (CC BY) license (http://creativecommons.org/licenses/by/4.0/).
